# A Collaborative Recommend Algorithm Based on Bipartite Community

**DOI:** 10.1155/2014/295931

**Published:** 2014-04-13

**Authors:** Yuchen Fu, Quan Liu, Zhiming Cui

**Affiliations:** ^1^Suzhou Industrial Park Institute of Services Outsourcing, Suzhou, Jiangsu 215123, China; ^2^School of Computer Science and Technology, Soochow University, Suzhou, Jiangsu 215006, China

## Abstract

The recommendation algorithm based on bipartite network is superior to traditional methods on accuracy and diversity, which proves that considering the network topology of recommendation systems could help us to improve recommendation results. However, existing algorithms mainly focus on the overall topology structure and those local characteristics could also play an important role in collaborative recommend processing. Therefore, on account of data characteristics and application requirements of collaborative recommend systems, we proposed a link community partitioning algorithm based on the label propagation and a collaborative recommendation algorithm based on the bipartite community. Then we designed numerical experiments to verify the algorithm validity under benchmark and real database.

## 1. Introduction 

Collaborative recommend technology is one of the most effective approaches in dealing with information overload. It has drawn great attention. However, with the development of network technologies, collaborative recommend has present complex dynamic characteristics and its key issues are still outstanding, such as data sparsity, scalable, and the shift of user interest.

In recent years, the study of complex networks has become a hot issue, and many theoretical models and analytical methods have been proposed. It provides new ideas and methods to solve those key problems. The bipartite network is an important manifestation of the complex network [1, 2], which could well portray collaborative recommendation system's topology. Therefore, scholars have begun to utilize these topologies to deal with the problems on collaborative recommendation [3–9]. These approaches could capture the global structure and the relation between the various basic elements, which avoid the impacts of ordinary objects, such as the popular resources and active users, and further improve the recommended results. In addition, these approaches could reduce requirements of original data. For privacy factors, available raw data is not rich, such as rating data and personal information. And analyzing text contents such as some comments is difficult, while those operating data, such as clicks and web retention time, are relatively readily available.

However, current research on collaborative recommend based on bipartite topology is just starting and mainly focuses on the global structure. It also contains a clustering based on certain pattern of users and resources, such as users' common interests and similar resources' theme. This local feature, known as the community structure in complex networks, is very beneficial to collaborative recommendation, including the following three points.

1. The communities are forming naturally and their size are controllable. Using them as nearest neighbourhoods can guarantee the relations between the object and its neighbours and enable the size of nearest neighbourhood to be dynamic. Compared with traditional methods, collaborative recommendation based on bipartite community can reduce influences of mistaken nearest neighbours. On the other hand, processing on local community structures can alleviate the scalability issues to a certain extent.
2. Structural properties of communities, such as overlap and hierarchy, could enrich the available information for collaborative recommend system. Studying on the inherent relationship between those structural properties and collaborative recommend process can bring new breakthroughs in data sparse and interpretability problems.
3. Research on community structure evolution can also grasp the dynamic nature of the recommendation system and reflect the behaviour of the continuous interactive and user feedback. So we can also discover certain patterns and predict the tendency of community structure, which could intelligentize collaborative recommend process.

Thus, we first proposed a bipartite community partitioning algorithm according to the real data environment of collaborative recommendation. And then we proposed a novel collaborative recommendation algorithm using these bipartite communities. Finally, we verify the validity of the algorithms by numerical experiments and analysed the phenomenon and reasons of the experimental results.

## 2. Related Research

### 2.1. Dynamic Nearest Neighbourhood Algorithm

In order to handle large-scale data set, the traditional researches are mainly focused on kNN methods, which predict recommendations by those historical choices of the target user's *k* similar users. But fixed value of *k* cannot satisfy different users' requirements. For example, if the number of similar users is less than *k*, the user neighbourhoods generated from traditional methods will include unsimilar individuals, which could affect the recommend accuracy. Therefore, the size of the neighbourhoods should have a dynamic adaptability.

Reference [10] proposed collaborative recommendation algorithm based on indeterminacy neighbourhood. It selected neighbourhoods and trust subgroups by setting some thresholds and introduces a harmonic parameter to integrate user-based and resource-based collaborative recommendation. On this basis, reference [11] introduced opinion mining technology and adds semantic similarity measure for comments dimensions.

Reference [12] first corrects the similarity between the target users and their nearest neighbour by an improved overlap factor and the attribute of target resource class. Then it predicted resources' scores to certain target user after rearranging the sequence of neatest neighbours. Finally it recommended resources by sorting these scores.

The above algorithms both adopted the idea of dynamic nearest neighbourhoods. The former focused on the neighbourhood scale which is suitable for the target user's forecast scenarios, while the latter's concern is the modification of neighbourhood based on the attribute of resource class. In addition, the former integrated user-based and resource-based collaborative recommendation. These algorithms overcome the limitations of traditional methods with fixed nearest neighbourhood and single dimension measure. But there are still some defects. The former needs to set a large number of parameters artificially, while the optimal value of parameter is difficult to determine under different scenarios, which will affect the algorithm stability. And resource class attribute introduced by the latter algorithm is too objective to reflect the rich content of users' subjective behaviour.

### 2.2. Bipartite Community Division

The division of bipartite community is the process of identify bipartite network community. It has important theoretical significance and practical value on network structure analysis, functional evolution, and prediction. In General, we can get the structure of bipartite community by project bipartite network for a common network of one kind nodes and execute existing community division algorithms. However, the projection process will result in loss of information and other issues. Therefore, many scholars directly divide bipartite community against the original bipartite network structure. Existing bipartite community methods generally fall into three categories: modular-based methods, clique-based methods, and propagation-based methods.

There are two main policies in modular-based methods. One is regarding the modular as the target function for optimization [13–16]; the other is regarding modular as a stop condition in the hierarchical clustering [17]. On one hand, the modular depends on global network and has a resolution limitation problem. On the other hand, heuristic searching is relatively complex and time consuming. Therefore, with high computational and time complexity, modular-based algorithms are not appropriate for large-scale real networks. In addition, the above algorithms are restricted to nonoverlap communities.

Clique-based methods divide overlapping bipartite communities. Reference [18] extended the *k*-clique community division algorithm in common network. It defined bipartite clique *K*_*a*,*b*_ as fully connected bipartite subgroup which consists of *a* *X* nodes and *b* *Y* nodes. And it defined the structure of bipartite clique community as a union of a series of adjacent bipartite clique which share *a*-1 *X* nodes and *b*-1 *Y* nodes at least. Then it implemented community division by the clique seepage. Reference [19] defined bipartite core sub-clique and executed the clustering method. Communities obtained by the above algorithms contain two kinds of nodes and have no heterogeneity.

Propagation-based methods are easy to implement parallel with a linear time complexity and without prior knowledge. Reference [20] introduced an improved label propagation algorithm for the bipartite network. It merely assigned different label to one certain kind of nodes at first, and then repeatedly synchronously updated the labels based on their heterogeneous neighbours in each iteration until a stable state was reached. However, this method is also restricted to nonoverlap communities.

### 2.3. Link Community Division

For users and resources are different objects, the bipartite communities under collaborative recommendation environment should be able to distinguish between heterogeneous nodes in order to guarantee interpretability. At the same time, due to the common phenomenon of milt-interested users and milt-theme resources, the overlap and hierarchy of bipartite communities should be allowed. The division of link community is an effective way to achieve the above targets. However, current researches on link community division are mainly in common network.

Reference [21] provided a general framework of link community division. The basic idea was to build a weighted edge graph for the bipartite network and then directly use existing community division algorithms. The method could adapt to the existing algorithms but needs extra time and memory consumptions for the edge graph. In addition, the edge graph of bipartite networks will produce two types of edges.

Reference [22] defined a similarity measure based on nonsharing vertexes of a pair of edges and utilized the simple hierarchical clustering to obtain a dendrogram of link communities. However, the clustering process and measurement are both depending on global information. On the other hand, the dendrogram consumed large storage space, and many of its levels did not have practical significance. Otherwise, link communities on different levels of the dendrogram is not in the general sense of multiscale but just a process of merging or splitting communities layer-by-layer.

According to the collaborative recommend systems' environments and requirements, our community division algorithm needs to implement the following goals except for the accuracy: (1) low complexity, computational complexity, and parallelizability for the large-scale data, (2) adaptability for dynamic updating data, and (3) overlapping, hierarchical, and related community structures for the multiple content data. Therefore, we draw on the idea of label propagation to divide link communities into bipartite network.

## 3. Methods

Without loss of generality, defining a bipartite network as *G* = (*V* ^*X*^, *V* ^*Y*^, *E*), where *V* ^*X*^ = {*x*_1_, *x*_2_,…, *x*_*n*_*X*__} is a set of nodes of kind *X*, *n*_*x*_ is the number of *X* nodes and *V* ^*Y*^ = {*y*_1_, *y*_2_,…, *y*_*n*_*Y*__} is a set of nodes of kind *Y*, *n*_*Y*_ is the number of *Y* nodes and *V* ^*X*^∩*V* ^*Y*^ = *∅*, *E*⊆(*V* ^*X*^ × *V* ^*Y*^). We note that the set of all nodes is *V* = *V* ^*X*^ ∪ *V* ^*Y*^, *n* is the number of nodes, and *m* is the number of links. We call the nodes of the same type as homogeneous nodes and those of the different types as heterogeneous nodes.

For a given node *i* ∈ *V*(*i*), we define its heterogeneous neighbour collection as *N*(*i*) = {*j* | (*i*, *j*) ∈ *E*, *j* ∈ *V* − *V*(*i*)}, which is a collection of nodes which directly connected to node *i*. Accordingly, it is the homogeneous neighbour collection a set of homogeneous nodes which shared heterogeneous neighbor with *i*, defined as Γ(*i*) = {*j* | *N*(*i*)∩*N*(*j*) ≠ *φ*, *j* ∈ *V*(*i*),  *j* ≠ *i*}.

### 3.1. Correlation Measurement

#### 3.1.1. Vertex Correlation

The heterogeneous neighbor is the direct property of a node; the degree of a node is the number of its heterogeneous neighbors, while the homogeneous neighbor is the indirect property of a node, because a node and its homogeneous neighbors contact with each other through those common heterogeneous neighbors. This interconnection is referred to as “cross linking,” and bipartite networks is exactly a cross-linked network. A Cross-linked structure is the basic unit of bipartite networks consisting of a pair of homogeneous nodes and common heterogeneous nodes. The formal definition is as follows.

**Definition 1**

In a given bipartite network *G*, nodes *i*, *j*, *k* form a cross-linked structure, if and only if they satisfy (*i*, *k*) ∈ *E*(*G*)∩(*j*, *k*) ∈ *E*(*G*). All the cross-linked structures which contain nodes *i* and *j* compose a cross-linked set (*i*, *j*). In other words, set (*i*, *j*) consisted of *i*,*j* and their common heterogeneous neighbors.

There is a certain correlation between a pair of homogeneous nodes in a cross-linked structure. For example, a cocitation relation means that literature articles are more or less similar to some degree if they have the same quotations. Therefore, reference [8] characterizes relations between homogeneous nodes by giving them cognitive ability and distinguishes their different position in the cross-linked structure. In this way, it completed the network projection and implemented collaborative recommendation based on the projection network. Combining with Definition 1, relevant formal definition is as follows.

**Definition 2**

Given a cross-linked structure *CrossLinkStructure*_〈*i*,*j*〉_(*k*), node *k* is referred to as the intermediary node, while node, *i* and *j* are referred to as subjective target node and objective target node, respectively. So, the *k*-intermediary cross-linked correlation that node *i* acts on *j* is

(1) $$\begin{array}{c} CL\_Correlation_{\langle i,j\rangle }\left(k\right)=f_{des}\left(k\right)f_{ind}\left(i\right)f_{\Delta }\left(j\right), \end{array}$$

where these three functions and their forms are defined as (1) the description ability of the intermediate node: *f*_*des*_(*k*) = 1/*k*_*k*_; (2) the independence of the subjective target node: *f*_*ind*_(*i*) = 1/*k*_*i*_; (3) the exclusiveness of the subjective target node to the object one: *f*_Δ_(*j*) = 1/(*k*_*j*_ − *m* + 1)^*α*^, where *α* is a parameter indicating degree of the contribution that the exclusiveness makes on the correlation and *m* = |*N*(*i*)∩*N*(*j*)|.

By accumulating the all correlations in cross-linked set (*i*, *j*), we can compute the correlation between target node *i* and its homogeneous neighbor *j*, expressed as 〈*i*, *j*〉 correlation. After normalization on all 〈*i*, *j*〉 correlations, we can obtain the importance probability 〈*i*, *j*〉 which means how important the node *j* is to node *i*. Relevant formal definitions are as follows.

**Definition 3**

Given a node *i* and one of its homogeneous neighbor node *j*, the 〈*i*, *j*〉 correlation is denoted as

(2) $$\begin{array}{c} clcorr_{\langle i,j\rangle }=f_{ind}\left(i\right)f_{\Delta }\left(j\right)\overset{}{\underset{k\in N\left(i\right)\cap N\left(j\right)}{\sum }}f_{des}\left(k\right). \end{array}$$

**Definition 4**

Given a node *i* and one of its homogeneous neighbor node *j*, the importance probability of *j* in the view of *i* is denoted as

(3) $$\begin{array}{c} \underset{j\in \Gamma (i)}{\sum }Sig_{\langle i,j\rangle }=\underset{j\in \Gamma (i)}{\sum }\tau \cdot clcorr_{\langle i,j\rangle }=1, \end{array}$$

where *τ* is normalization factor.

**Figure 1:**
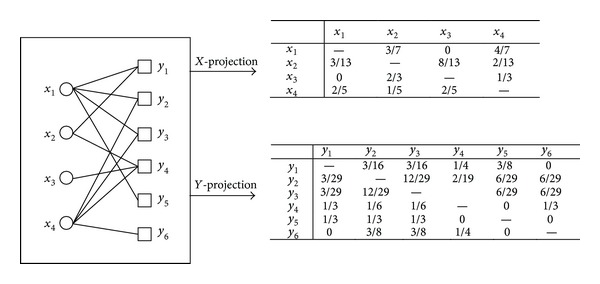
An example of calculating vertex correlations. The left-hand figure is the structure of a bipartite network and the right-hand one shows correlation matrixes.

Figure 1 shows an example on calculating vertex correlations according to the above definitions where the value of *α* is 1.

#### 3.1.2. Edge Adjacent Structure

In a common network, we generally say that two edges sharing the same endpoint are adjacent, because these edges clearly have higher similarity than those without common endpoint. But the common endpoint is unable to provide useful information for the similarity measure, and the higher its degree is, the more similar the edges are. Therefore, Ahn et al. [22] used the modified Jaccard index based on nonshared endpoints to measure the similarity of adjacent edges.

However, an edge exists in a pair of heterogeneous nodes in bipartite network. So, the common nodes of adjacent edges are different types.

For example, in Figure 1 adjacent edges of edge (*x*_1_, *y*_1_) are (*x*_1_, *y*_2_), (*x*_1_, *y*_3_), and (*x*_2_, *y*_1_). The common node shared by (*x*_1_, *y*_1_) and the former two edges are of type *X*, while the node which (*x*_1_, *y*_1_) shared with the last one is of type *Y*. If we follow Ahn's similarity measure, the similarity between edge (*x*_1_, *y*_1_) (*x*_1_, *y*_2_) and (*x*_1_, *y*_3_) is represented by the similarity between *Y* nodes *y*_1_(*y*_2_) and *y*_3_, while the similarity between edges (*x*_1_, *y*_1_) and (*x*_2_, *y*_1_) is represented by the similarity between *X* nodes *x*_1_ and *x*_2_. This results in the inconsistent similarity measure, and we can judge which one of the adjacent edges is more related to (*x*_1_, *y*_1_).

Therefore, for a given edge, we define another edge as its adjacent edge if and only if the edge has no common endpoints with it and each pair of homogeneous endpoints of them has common heterogeneous neighbors. This could unify the correlation of both dimensions, and we could measure it by the correlations of the two pairs of homogeneous nodes. For example, in Figure 1 the set of adjacent edges of edge (*x*_1_, *y*_1_) is {(*x*_2_, *x*_4_), (*x*_4_, *X*_2_), (*x*_4_, *x*_3_), (*x*_4_, *x*_4_)}, relevant formal definition is as follows.

**Definition 5**

Two edges are adjacent if and only if they have no common endpoints and each pair of homogeneous endpoints shares common heterogeneous neighbors. For a given edge (*i*, *l*), its adjacent edges set is

(4) Ne(i,l)={(j,m) ∣ i≠j,l≠m,N(i)∩N(j)≠∅,N(l)∩N(m)≠∅}.

**Corollary 6**

If edge (*i*, *l*) is adjacent to (*j*, *m*), then common homogeneous neighbors of node *l* and *m* are containing common heterogeneous neighbors of node *i* and *j*, meaning (*i*)∩*N*(*j*)⊆Γ(*l*)∩Γ(*m*).

#### 3.1.3. Edge Correlation

The property of an edge is determined by its endpoints. Therefore, we can measure the correlation of adjacent edges through indirectly multiplying the correlations of homogeneous endpoints. Relevant formal definition is as follows.

**Definition 7**

Given a pair of adjacent edges (*i*, *l*) and (*j*, *m*), their correlation under (*i*, *l*)'s perspective is denoted as

(5) $$\begin{array}{c} ecorr_{\langle e_{il},e_{jm}\rangle }=Sig_{\langle i,j\rangle }\cdot Sig_{\langle l,m\rangle }. \end{array}$$

The above definition uses the correlation between two pairs of homogeneous neighbor nodes independently. When calculating Sig 〈*i*, *j*〉, each intermediary node of nodes *i* and *j* contributes to the correlation equally.  Corollary 6 shows that intermediary nodes are also the common homogeneous neighbors of nodes *i* and *m*. Therefore, for an intermediary node, its ability to depict the correlation of the pair of homogeneous endpoints *I* and *j* becomes greater, if and only if the correlation between *l* and it is closer to the one between *m* and it. After modifying the description ability of intermediate nodes, we could obtain the correlation 〈*i*, *j* | *l*, *m*〉; the amended definition is as follows.

**Definition 8**

For a given pair of adjacent edges (*i*, *l*) and (*j*, *m*), their correlation under (*i*, *l*)'s perspective is

(6) $$\begin{array}{c} ecorr_{\langle e_{il},e_{jm}\rangle }=Corr_{\langle i,j\rangle }\left(l,m\right)\cdot Corr_{\langle l,m\rangle }\left(i,j\right), \end{array}$$

where *Corr*_〈*i*,*j*〉_(*l*, *m*) represents the 〈*i*, *j* | *l*, *m*〉 correlation, and it satisfies

(7) Corr〈i,j〉(l,m) =∑k∈N(i)∩N(j)Sim(Sig〈l,k〉,Sig〈m,k〉)CLCorrelation〈i,j〉(k),

where *Sim*(*Sig*_〈*l*,*k*〉_, *Sig*_〈*m*,*k*〉_) represents the correction factor denoting how much the contribution intermediary node *k* in cross-linked structure (*i*, *j* ) made to the 〈*i*, *j* | *l*, *m*〉 correlation, and it satisfies

(8) ∑k∈N(i)∩N(j)Sim(Sig〈l,k〉,Sig〈m,k〉)  =∑k∈N(i)∩N(j)τ·1|Sig<>〈l,k〉−Sig〈m,k〉|+1=1.

If the intermediary node *k* is exactly one endpoint of the edge, the correction factor is 1. It is like that people think their own ideas most important.

Table 1 shows an example of calculating correlations among adjacent edges.

### 3.2. Bipartite Community Division Algorithm Based on Propagation

In the real world, people often follow others' behaviors. For example, they will buy the same goods that their friends have bought. The edge correlation measure proposed in last section could be interpreted as to how a behavior depends on another one. Here, we propose a link community division algorithm based on label propagation (BELPA).

#### 3.2.1. Algorithm Design

The basic idea of BELPA is assigning unique labels to each edge at first and then repeatedly updating labels until they converge to a steady state. At last, edges with the same label belong to the same community. This process is equivalent to label propagation on a directed and weighted network where nodes are corresponding to edges in the bipartite network and the directed and weighted edges are corresponding to the correlation between adjacent edges. We need to solve three key problems: how to allocate initial labels, how to update labels, and when to stop the iterative process.

First of all, we select one kind of node sets as the starting set and then give the same label for edges ending up with each node in the node set. For example, if starting from the set of *X* nodes, the label assigned to each edge is the identification of the edge's *X* endpoint. This process is consistent with the idea in [19], because it is equivalent to the idea that *Y* nodes can also receive the labels from *X* nodes.

After initial allocation, the label updating strategy includes following aspects.

(1) Label selection strategy. At the *t*th iteration, we weight the correlation between a given edge *α* and its adjacent edges and update its own label by adjacent label with the highest correlations. The label updating function is
  (9) $$\begin{array}{c} C_{\alpha }\left(t\right)=\underset{C}{\arg\;\max}\underset{\beta \in Ne\left(\alpha \right)}{\sum }ecorr_{\langle \alpha ,\beta \rangle }\left[C_{\beta }\left(t-1\right)==C\right]. \end{array}$$
(2) Tie treatment strategy. When the above function returns more than one maximum labels, we will maintain the label of edge *α* if it is one of them, otherwise we select one of them randomly.
(3) Updating execution strategy. We execute label updating synchronously, that is, the new label of each edge is independent of other edges in the current iteration and just relies on the adjacent labels in the last iteration. So we can obtain more stable results and make the algorithm parallel and practical.

Finally, in *t*th iteration, if one of the following conditions is achieved, we will stop the algorithm:

1. no edge updates label, namely, *C* (*t*) = *C*(*t* − 1);
2. after updating, labels satisfy the condition *C* (*t*) = *C*(*t* − 2);
3. the maximum iteration is reached, namely, *t* > *T*; *T* is set previously.

According to the above steps, we execute the edge label propagation on the bipartite network shown in Figure 1, and the process is shown in Table 2.

In the initial iteration (iteration zero), the labels are identifiers of *X* nodes. Finally we can obtain two *X* communities: {*x*_1_, *x*_2_} and {*x*_2_, *x*_3_, *x*_4_}, and two *Y* communities: {*y*_1_, *y*_2_, *y*_3_, *y*_5_} and {*y*_2_, *y*_4_, *y*_6_, *y*_3_}. Node *x*_2_ is the overlap section in *X* communities, and both forms of its community membership are 0.5. Nodes *y*_2_ and *y*_3_ are the overlap section in *Y* communities, and both forms of their community membership are also 0.5. Furthermore, community identifiers are 1 and 4, respectively, which are initial labels reserved at last. Nodes *x*_1_ and *x*_4_ have higher degree and could make stronger influence on others so they become the community core and the initial label from them was reserved. On the contrary, nodes *x*_2_, *y*_2_, and *y*_3_ are all connecting the heterogeneous nodes with high degree, so they are receiving comparative attractions from two communities and become community border.

#### 3.2.2. Algorithm Expansion

Radicchi et al. [23] proposed the comparative definition of community dividing community into strong community and weak community. The strong community focuses on each node in it, while the weak one focuses on the whole community. On the other hand, the definition is still based on link density, namely, that links are dense in communities and are sparse among communities. In bipartite networks, link pattern is more appropriate, because no links exist in and among homogeneous communities and meaningful bipartite community only contains homogeneous nodes. Because the correlation measure in upper section is calculated indirectly according to the network topology, which is equivalent to be obtained based on some kind of link pattern, it will be effective to divide community by our correlation definition.

The hierarchical clustering algorithm [21] deals with edges in the whole network layer-by-layer and then gets different-scale communities by cutting the dendrogram. Communities on different levels of the dendrogram constitute the community hierarchy. The closer to the top level of the dendrogramis, the larger community will be obtained. However, this hierarchy is exactly equivalent to the different period during the merging and disintegrating communities; the optimal value will appear on a certain level of the dendrogram under the rule of cutting to meet biggest modular. So we take example by the definition of strong and weak community and introduce a parameter to control the label propagation process so that we can get a community of different scale.

That the given edge completes its label updating is equivalent to saying that the edge has joined into a link community with its new label. And this change will make an effect on the original link community, because the correlation between adjacent edges is bidirectional. For example, the whole correlation in the link community will be weakened, if the correlations between original edges inside and the given edge are very weak. This is similar to the access permission of some organizations in real life, such as some people who will be rejected to join in. Therefore, we modify formula (9) as

(10) Ca(t)=arg max⁡C(t−1)(∑eβ∈EN(eα)ecorr〈α,β〉−γ·(ecorr〈α,β〉−ecorr〈β,α〉)).

Here, parameter *γ* denotes a factor of influence of how many members in the community will accept others out of the community. When *γ* = 0, it is just considering correlations under the view of the given edge itself. When *γ* = 1, it goes to the other extreme, namely considering correlations under the view of adjacent edges only. We can get different-scale communities by adjusting value of *γ* and Table 3 shows a simple example. From the table, we can see that some smaller communities are recognized when the parameter grows, which will be helpful to find out nested structures.

### 3.3. Recommend Algorithm Based on Bipartite Community

We utilize BELPA algorithm to obtain the user and resource community and then forecast the value of resources which are not chosen by target users according to the community membership and corresponding relationship between communities to realize collaborative recommendation.

#### 3.3.1. Related Concepts

Firstly, we take user and resource community as users' and resources' nearest neighbourhood, respectively, and call those community members as users' and resources' community neighbors, respectively. For a given user *u* and a given resource *r*, the formal definitions of their community neighbors are Γ_*C*_^*U*^(*u*) = {*v* | *v* ∈ *C*(*u*)} and Γ_*C*_^*R*^(*r*) = {*k* | *k* ∈ *C*(*r*)}, where *C*(*u*) and *C*(*r*) represent the community to which user *u* and resource *r* are belonging.

Secondly, we call those heterogeneous communities with same community label as corresponding community; that is, for any node ∈*V*(*u*), if it belongs to the community *C*_*i*_^*V*(*u*)^, its corresponding community is *C*_*i*_^*V*−*V*(*u*)^. In particular, we define *C*_*ur*_ as the corresponding community for user *u* and resource *r*. Thus it can be seen that the membership of node *u* in community *C*_*i*_ has two meanings: one is the participative extent of user *u* in community *C*_*i*_^*V*(*u*)^ and the other is the extent of how important community *C*_*i*_^*V*−*V*(*u*)^ is to user *u*. So, we take the community memberships as:

1. a weighting coefficient which is used to calculate correlations between a given user or resource and their community neighbors;
2. an initial score that a given user gives to his selected resource.

Finally, we use cosine theorem to calculate the similarity based on the community membership. The formal definition is as follows.

**Definition 9**

The similarity based on community membership of given node *u*, *v* ∈ *V* ^*U*^ ∪ *u*, *v* ∈ *V* ^*R*^ is

(11) cbsim(u,v)  =∑Ci∈C(u)∩C(v)u.mem(Ci)·v.mem(Ci)∑Ci∈C(u)u.mem(Ci)2·∑Ci∈C(v)v.mem(Ci)2,

where *u*.*mem* (*C*_*i*_) and *v*.*mem* (*C*_*i*_) represent membership of nodes *u* and *v* in community *C*_*i*_, respectively.

Combining with the above definition, we use larger value strategy to modify the correlation measure between target object and its community neighbor, and weighting by its community membership. So, the correlation between given object and its community neighbor is defined as follows.

**Definition 10**

The correlation between given node *u* and its community neighbors is defined as

(12) ∑v∈ΓC(u)cbcorr〈u,v〉  =∑v∈ΓC(u)τ·[max⁡(cbsim(u,v),clcorr〈u,v〉)·∑Ci∈C(u)∩C(v)u.mem(Ci)]=1.

#### 3.3.2. Algorithm Design

We maintain a recommended list *rl* for a target user *u*; the element in the list consists of the resource identifier and the score that user *u* may rate in the resource. The resource with the higher score will be prior to be recommended to users. The recommended list is empty initially.

For a target user *u*, the steps of user-community-based collaborative recommend (UCBCR) algorithm is as follows:

(1) determining the user community neighbourhood Γ_*C*_^*U*^(*u*) for *u*;
(2) for ∀*v* ∈ Γ_*C*_^*U*^(*u*), adding the resources which have been chosen by *v* but not been selected by *u* to *rl*, and accumulating their scores brought from *v*. The score of resource *r* brought from user *v* is defined as
  (13) $$\begin{array}{c} score_{v}\left(r\right)=cbcorr_{\langle u,v\rangle }\cdot v.mem\left(C_{vr}\right), \end{array}$$
  where *v*.*mem*(*C*_*vr*_) denotes the initial score of resource *r* rated by user *v*;
(3) sorting resources in the *r*_*l*_ according to their scores.

Similarly, the steps of resource-community-based collaborative recommend (RCBCR) algorithm is as follows:

(1) for ∀*k* ∈ *N*(*u*), determining the resource community neighborhood Γ_*C*_^*R*^(*k*) for *k*;
(2) adding resources in Γ_*C*_^*R*^(*k*) which have not been selected by *u* to *rl* and accumulating their scores brought from community neighbor *k*. The score of resource *r* brought from resource *k* is defined as
  (14) $$\begin{array}{c} score_{k}\left(r\right)=cbcorr_{\langle k,r\rangle }\cdot u.mem\left(C_{uk}\right), \end{array}$$
  where *u*.*mem*(*C*_*uk*_) denotes the initial score of resource *k* rated by *u*;
(3) sorting resources in the *rl* according to their scores.

## 4. Numerical Experiments

### 4.1. Experiments of Community Division Algorithm

Standard data set. We used Southern Women data set to verify the validity of BELPA algorithm in this section. This data set describes the participation of 18 women in 14 social events. Many social scientists have divided 18 women into two groups: woman from 1 to 9 and woman from 10 to 18. Some other social scientists think that woman 9 belongs to both groups. Generally, the real women community partition of this data set is expressed by {1 ~ 9} and {10  to 18}.

Table 4 shows different results of related algorithms. We can see that the algorithm results of Guimerà et al. [13] are the optimal compared with the real partition. The algorithm results of Barber [14] misclassify woman 8, and algorithm results of Murata [15] and Suzuki and Wakita [16] contain many isolated small communities, especially Suzuki's. These differences are due to the format of bipartite modularity, especially that Murata and Suzuki both design modularity to find communities with many-to-many relationships. It also can be speculation that the origin of these differences is the different positions of nodes in bipartite network.

Therefore, we deem that the reasonable result contains two parts: the foundation partition which consists of two communities {1 ~ 9} and {10 ~ 18} and other small communities which are overlaps among communities, such as {8}, {9}, and {16}. What is more, members in the overlaps should be more affiliated to its foundation partition. For instance, communities {8}, {9} should be more affiliated to the community {1 ~ 9} and {16} should be more affiliated to the community {10 ~ 18}. This reasonable result will be more credible and has practical significance in the real world.

Table 5 shows the result of BELPA algorithm. When *γ* is ranging from 0 to 0.4, woman 16 is the overlap in women communities and its membership of community {10 ~ 18} is 0.5. When *γ* reaches 0.5, we can get the same result with the real partition. When *γ* is ranging from 0.6 to 1, woman 8 is the overlap and its membership of community {1 ~ 9} is 2/3. When *γ* is ranging from 0.8 to 1, woman 9 is the overlap and its membership community of {1 ~ 9} is 3/4.

We can also see that the final community label is 1 and 13, because the two women 1 and 13 have high frequency to participate in social events and they become the core of communities. Then, women 16, 8, and 9 nodes in overlaps all have lower frequentness of participating in social events, 2, 3, and 4, respectively, and they all have taken part in two social events, 8 and 9, in which many women have participated. So they are pulled by two communities and become community border.

The above experiments show that BELPA could obtain reasonable results consistent with the real partition under different parameter values. What is more, BELPA could identify the cores and borders of communities which are representatives of communities and bridges connecting different communities, respectively. This will play an important role in the practical applications.

Only meaningful communities in the real world can be further used of to achieve collaborative recommendation. Therefore, in this section we use MovieLens data set to verify the validity of BELPA algorithm. In order to test the recommendation algorithms afterwards, we extract some data in a random time slot from the original data set and divide it into two parts on the basis of time sequence; the training set contains 80% of it and the test set contains 20% of it.

**Figure 2:**
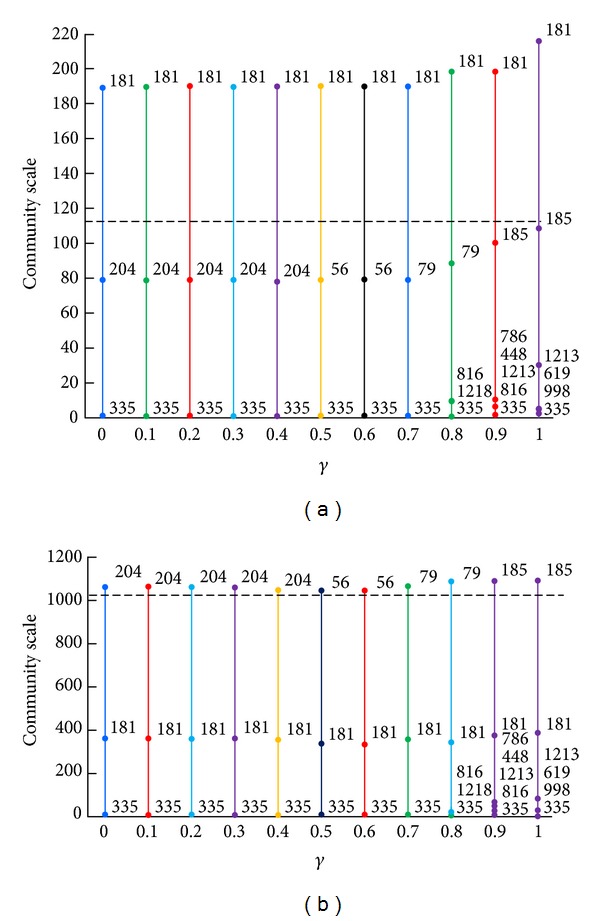
The community division result of MovieLens data set starting from resource nodes. The abscissa and ordinate denote the parameter value and community scale, respectively. Figure at left shows user communities and the one at right shows resource communities. The dotted horizontal lines show the number of users and resources, respectively, and solid vertical lines represent community distributions. Each segment of the solid line shows a community and the length denotes community size. The labels beside nodes are the identifiers of communities.

The data set in this experiment contains 113 user nodes and 1024 resource nodes. The average degree of user nodes is 70 and the average degree of resource nodes is 7.8. If we choose resource nodes as the starting set, we can finally obtain the result shown in Figure 2. We can get the following conclusions.

1. Basic community structure. With the growth of parameter *γ*, the data set was divided into three basic communities and the number of communities increased slightly along with the appearance of some small communities. Some community identifiers changed, which showed that internal structure of community including its core and border was changing. The 181 and 335 community are two rather stable communities among them. The 181 resource has high degree, so it becomes the core of community and has great influence on other nodes. This kind of resource often becomes the main focus in the real world. On the other hand, the degree of 335 resource is 2 and it connects 130 user whose degree is 1, which results in the edge between them having no adjacent edges and becoming an isolated community. In addition, some nodes with medium degree become the core of communities alternately, such as 204, 56, 79, and 185.
2. Nested community structure. With the growth of parameter *γ*, some small communities are separated, such as 816, 1213, and 619. Because the degrees of these resource nodes are all 2 and they are connected to two user nodes with higher degree, which makes them be pulled by the two link communities having comparative attraction at the same time, those resource nodes will be finally separated as special bordered structure of community. In other words, these small communities are not the isolated structures but the nested structures, which is meaningful in practical applications. It is like that there always exist smaller groups with closer relationships in a large group.
3. Overlapping degree. The length of solid vertical lines beyond the dotted horizontal line shows the overlapping degree of communities. So we can see that the overlapping degree of user community is bigger than the one of resource community, because the average degree of user node is bigger, namely, that user nodes ending up with more edges will be more likely to belong to different communities.

**Figure 3:**
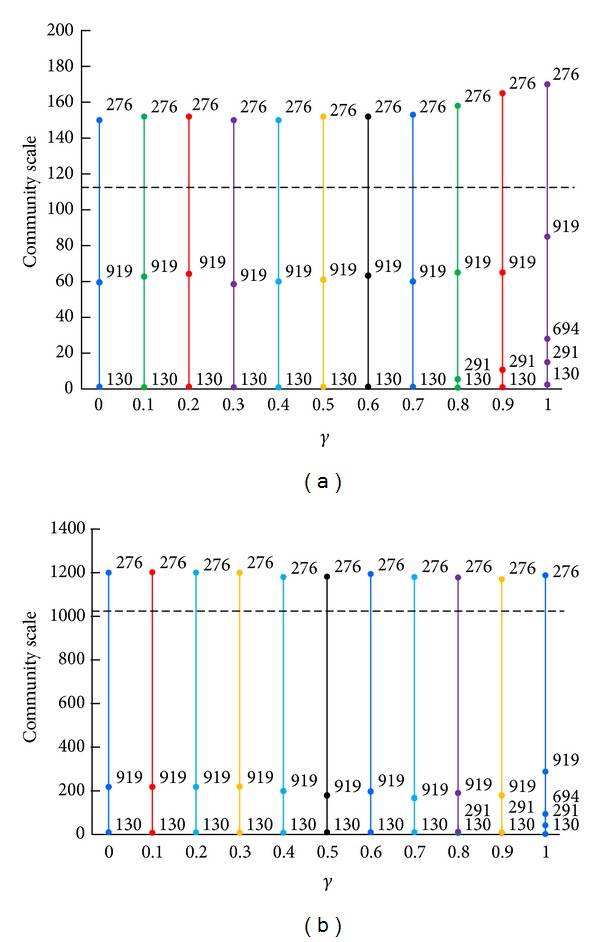
The community division result of MovieLens data set starting from user nodes. Related instructions are shown in Figure 2.

Starting from user nodes, we can finally obtain the results shown in Figure 3. Comparing results in Figures 2 and 3, we can get the following conclusions.

1. There are slight differences in both the number and size of communities between basic partitions of them. And there is also a certain correspondence between them. For example, 130 community is separated because it is connected to 335 resource.
2. The former user communities are more overlapped than the latter ones, while the former resource communities are less overlapped than the latter ones. In the first iteration, nodes in the staring set can gain only one initial community label, while nodes in the receiving set can get more than one label through edges of different labels. Finally the former user communities with user nodes as receivers are more overlapped than the latter ones with user nodes as senders and so does the situation of rescores nodes.
3. The former nested community structures are slightly more than the latter ones with the growth of the parameter value, because the scale of resources in this experimental data set is larger than the scale of users, which makes the former number of initial labels slightly more bigger and nested community be easier to separate.

Above all, BELPA algorithm can get bipartite communities in the real data set. What's more, it can identify cores borders and of communities and some nested structures, which will play an important role in collaborative recommendation systems. For instance, we can use community cores to ease cold start-up problem or use community borders and nested structures to improve the recommendation diversity. Moreover, by comparing the results of different initial label distribution strategy, we found that the results of BELPA algorithm are relatively stable. But specific initial label distribution strategy and data structure will both affect the division results indeed.

### 4.2. Experiment of Collaborative Recommend Algorithm

#### 4.2.1. Evaluating Measures

Measures of collaborative recommendation contain accuracy and individualization. The former is the degree of correspondence between recommend results and users' preferences, and the latter is the difference degree between recommend results of different users.

We use rank accuracy and hitting rate to measure the accuracy. Rank accuracy is the mean of ranking score, which is defined as

(15) $$\begin{array}{c} r_{i}=\frac{L_{i}}{N}, \end{array}$$

where *i* is a resource in the testing set, *N* is the number of resources which is not chosen by a certain user in the training set, and *L*_*i*_ is the position of resource *i* in the recommendation list. Smaller the *r*_*i*_ value is, higher the chosen resource is likely to rank in the list of recommendation, in brief, more accurate of algorithm.

In fact, users only concern resources at the front of recommend lists, so we use hitting rate to measure the percentage that the number of resources chose by the target user to a certain list length with certain length list. The hitting rate is defined as

(16) $$\begin{array}{c} h_{i}=\frac{L_{c}}{L}, \end{array}$$

where *L* is length of recommend list and *L*_*c*_ is the number of resources that appear in both the testing set and the recommendation list. The higher the hitting rate is, higher the algorithm accuracy is.

We use popularity and diversity to measure the individualization degree of algorithm. The popularity is measured by average degree. In the real world, if the recommended results contain many popular resources which are chosen by many users, the accuracy could be guaranteed, while the individualization perhaps could be weakened, because the popular resource may not meet the individual needs of users. Therefore, the smaller the average degree is, the more personalized the recommendation results are.

Hamming distance can measure difference degree between recommend lists. It is defined as

(17) $$\begin{array}{c} H_{ij}=1-\frac{Q_{ij}}{L}, \end{array}$$

where *L* is length of the recommendation list and *Q*_*ij*_ is the number of common resources between recommend list of user *i* and *j*. The diversity is just the average of the hamming distance on all recommended results, and the greater the average hamming distance is value, the greater the diversity is.

#### 4.2.2. Numerical Results

**Figure 4:**
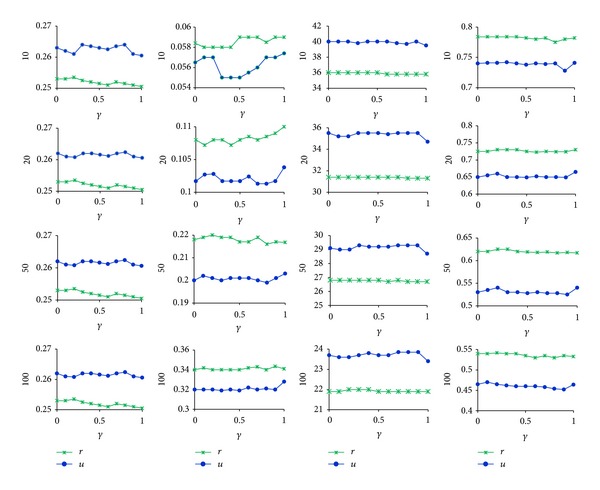
Results of UCBCR. It successively reports the rank accuracy, hitting rate, popularity, and diversity base on different length of recommend lists and different community structures under each parameter values. Illustrations *u* and *r* express the results base on user communities in Figures 2 and 3, respectively.

**Figure 5:**
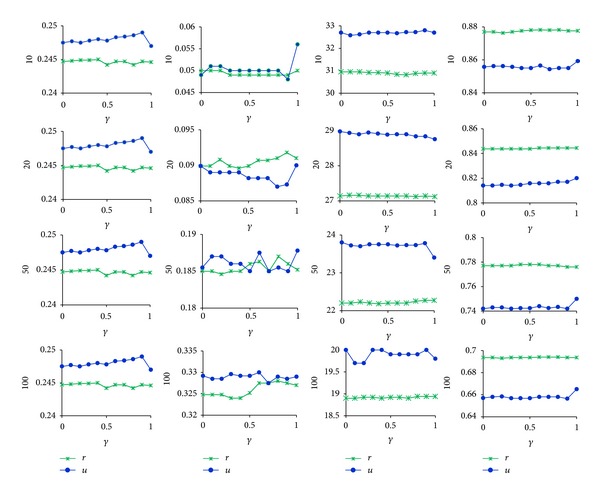
Results of RCBCR. Related instructions are shown in Figure 4.

In this section we used the above measures to estimate our collaborative recommend algorithm based on communities in Figures 2 and 3. The results of UCBCR and RCBCR are shown in Figures 4 and 5, respectively.

Looking at the overall trend of different indexes in the figures, we can get the following conclusions.

1. Each value of different measures under different parameter values presented a gentle change, which verified that the basic partition changed both on size and number of communities as we have mentioned before.
2. The variation tend of these measure values keeps basic consistent with the one of both the overlapping degree of community structure and separation degree of nested structure in Figures 4 and 5. This shows that the greater the overlapping degree is or the more the nested structures are, the better the recommend effect is.

Different initial label assignment strategy results in different community partition. Therefore, observing results of illustrations *u* and *r* in Figures 4 and 5, we can get the following conclusions.

1. As a whole, the effects of UCBCR and RCBCR algorithm based on communities obtained by BELPA starting from user nodes are superior to the ones based on communities obtained by BELPA starting from resource nodes.
2. When the length of list is separately 10, 50, and 100, the hitting rate of RCBCR algorithm based on communities obtained by BELPA starting from resource nodes is higher than the one based on communities obtained by BELPA starting from user nodes. However, the diversity and popularity index is still slightly optimal in RCBCR algorithm based on communities obtained by BELPA starting from resource nodes.

As mentioned before, both user and resource communities obtained by BELPA starting from resource nodes are more meticulous, but the overlapping degree of resources communities is slightly lower than those obtained by BELPA starting from user nodes. Therefore, the above phenomenon can also explain that, the higher overlapping degree and the more apparent nested structure are helpful to improve recommend accuracy on the whole and guarantee individuation of algorithm at the same time.

In addition, there is an obvious inflection point on the curve of the illustration *u* in Figures 4 and 5 when parameter *γ* reaches 1, and each index value has a significant improvement. From Figure 5, we can find that, at this time, overlapping degrees of user communities and resources communities and the number of nested structures were obviously increased. This phenomenon again proves that the high overlap degree and apparent nested structure could improve the recommend effects on the whole.

The above analysis shows the relationship between effects of collaborative recommend algorithms and community characters, including the overlapping degree and the separation degree of nested structures. The reasons are mainly the following two points.

1. Because our collaborative recommend algorithms adopt the similarity based on the community membership, the higher the overlapping degree of communities is, the more abundant the information of nodes' community membership is. And we can depict the scale of neatest neighborhood and the correlations among the neighbors much more accurately.
2. The separation of nested structures comes down to find out those nodes as the community borders, which are bridges between different communities. They can expand the scope of the nearest neighborhoods by introducing other possible related objects, which could ensure both algorithms accuracy and recommend diversity, especially the latter.

**Figure 6:**
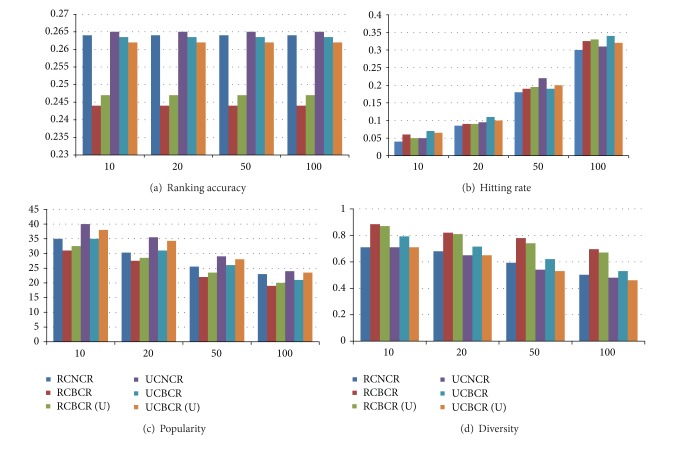
Results of different algorithms under some typical list lengths. (a), (b), (c), and (d) report the ranking accuracy, hitting rate, popularity, and diversity, respectively. The illustrations of RCBCR and UCBCR show the mean of all metrics UCBCR and RCBCR algorithms based on communities in Figure 4. And the illustrations of RCBCR (U) and UCBCR (U) show those results based on communities in Figure 5.

Finally, we compared metrics of our collaborative recommend algorithms ( UCBCR and RCBCR) with those of algorithms (RCNCR and UCNCR) as benchmark algorithms in [8]. The results are shown in Figure 6.

Analyzing the above results, we can get the following conclusions.

1. Compared with benchmark algorithms, each measure of UCBCR and RCBCR algorithms improved. Our algorithms ensured the algorithm accuracy and showed an obvious advantage in the recommend diversity individuation. It also proved that those user and resource communities could effectively represent the nearest neighborhood, which could verify the validity of BELPA algorithm at the same time. On the other hand, the community neighbors could break limits from cross-linked structure, which played an important role to raise the novelty of collaborative recommendations.
2. Comparing the results of different algorithms under different community divisions, the improvements of the algorithms UCBCR and RCBCR are more apparent than the ones of the algorithms UCNCR (U) and RCBCR (U), which we have mentioned before.
3. Comparing the results of different measures, on the algorithm accuracy, the improvement of the algorithm UCBCR is more apparent than the algorithm RCBCR, which verifies the importance of the community overlapping degree to improve recommend effects. On the recommend diversity, the improvement of algorithm RCBCR is very apparent, because the number of resources community is larger and the resources nodes in the community border can introduce new objects from other communities, so more community neighbors could become recommended objects. Another possibility is that if the overlapping degree is too high, it may reduce the recommend diversity.

## 5. Conclusion

In order to use local characters of the topology structure of collaborative recommend systems, we put forward a bipartite link community division algorithm based on the label propagation (BELPA). We redefined the structure of adjacent edges and the edge correlation measure by making full use of the properties of endpoints on the edge. Then we gave a label to each edge and synchronously updated labels according to edge correlations until steady state was reached. Those edges with the same label comprise a community. Taking example by the idea of defining strength and weak community, we expanded the basic algorithm by adjusting the label updating function to make the scale of community variable. Finally, we designed numerical experiments on relevant data sets to verify the algorithm validity.

We proposed a collaborative recommendation algorithm based on the bipartite community obtained by BELPA. In detail, we used the overlaps and corresponding relationship of the user resource communities to realize the dynamic nearest neighbourhood. At last, by the numerical experiment and the analysis of experimental results, we prove that our recommend algorithms could effectively improve the recommended accuracy and individuation.

## Figures and Tables

**Table 1:** An example of calculating the correlations among adjacent edges, where *α* is 0.5. Elements in this figure are normalized results.

	(1, 1)	(1, 2)	(1, 3)	(1, 5)	(2, 1)	(2, 4)	(3, 4)	(4, 2)	(4, 3)	(4, 4)	(4, 6)
(1, 1)	—	0	0	0	0	0.141	0	0.174	0.174	0.510	0
(1, 2)	0	—	0	0	0.673	0.099	0	0	0.415	0.140	0.278
(1, 3)	0	0	—	0	0.673	0.099	0	0.415	0	0.140	0.278
(1, 5)	0	0	0	—	0.175	0	0	0.413	0.413	0	0
(2, 1)	0	0.095	0.095	0.151	—	0	0.272	0.139	0.139	0.107	0
(2, 4)	0.289	0.221	0.221	0	0	—	0	0.118	0.076	0	0.118
(3, 4)	0	0	0	0	0.501	0	—	0.140	0.140	0	0.219
(4, 2)	0.158	0	0.410	0.248	0.097	0.033	0.053	—	0	0	0
(4, 3)	0.158	0.410	0	0.248	0.097	0.033	0.053	0	—	0	0
(4, 4)	0.158	0.170	0.170	0	0.092	0	0	0	0	—	0
(4, 6)	0	0.401	0.401	0	0	0.077	0.121	0	0	0	—

**Table 2:** An example of edge label propagation. The row numbers express edges, the column numbers express iteration times, and elements express edge label identifier.

	(1, 1)	(1, 2)	(1, 3)	(1, 5)	(2, 1)	(2, 4)	(3, 4)	(4, 2)	(4, 3)	(4, 4)	(4, 6)
0	1	1	1	1	2	2	3	4	4	4	4
1	4	4	4	4	4	1	2	1	1	1	1
2	1	1	1	1	1	4	1	4	4	4	4
3	4	4	4	4	4	1	4	1	1	1	1
4	1	1	1	1	1	4	1	4	4	4	4

**Table 3:** An example of getting different-scale communities by adjusting *γ* with step length 0.1.

*γ*	*X* style community	*Y* style community
0~0.4	{1, 2}, {2, 3, 4}	{1, 2, 3, 5}, {2, 3, 4, 6}
0.5~1.0	{2, 4}, {2}, {3}, {1}	{1, 2, 3, 5}, {2, 3, 4, 6}, {1}, {4}

**Table 4:** Results of related algorithm.

	Women community	Events community
Guimerà	{1~9}, {10~18}	{1~8}, {9~14}
Barber	{1~7, 9}, {8, 10~18}	{1~8}, {9~14}
Murata	{1~6}, {7, 9, 10}, {8, 16~18}, {11~14}	{1~6}, {7, 8}, {9, 11}, {10~14}
Suzuki	{1~7}, {8}, {9}, {16}, {17, 18}, {10~14}	{1~6}, {7}, {8}, {9, 11}, {10, 12~14}

**Table 5:** The result of BELPA algorithm starting from women set with 0.1 as the step length of parameter *γ*.

Γ	Women community	Events community
0~0.4	{1~9, 16}, {10~18}	{1~9}, {6~14}
0.5	{1~9}, {10~18}	{1~9}, {6~14}
0.6~0.7	{1~9}, {8, 10~18}	{1~9}, {6~14}
0.8~1.0	{1~9}, {8~18}	{1~9}, {6~14}
